# Diagnosis and Treatment of Rare Complication after Endomyocardial Biopsy

**DOI:** 10.5935/abc.20170120

**Published:** 2017-12

**Authors:** Viviane Tiemi Hotta, Daniella Diniz do Nascimento Rangel, Glaucia Maria Penha Tavares, Sandrigo Mangini, Pedro A. Lemos

**Affiliations:** Instituto do Coração (InCor) - Faculdade de Medicina da Universidade de São Paulo, São Paulo, SP - Brazil

**Keywords:** Heart Transplantation, Graft Rejection, Biopsy, Arterial Fistula/surgery, Echocardiography

**Figure 1:**
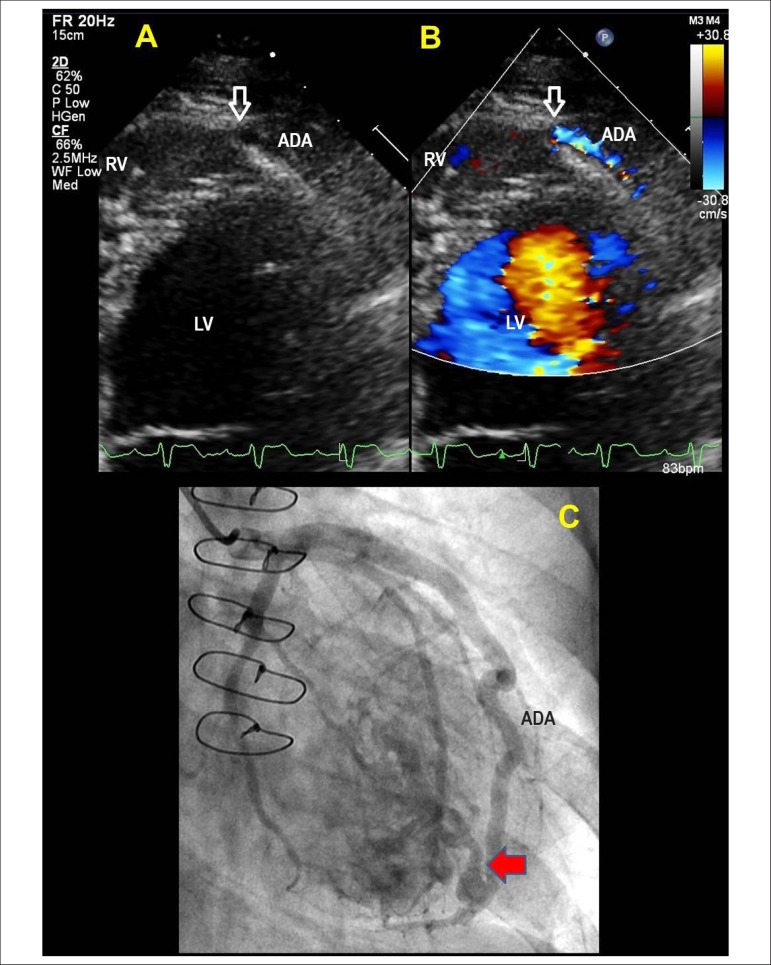
Images obtained from two-dimensional echocardiogram from the apical four-chamber zoom (A) and color Doppler study (B) demonstrating coronary-cavitary fistula (arrow) from ADA to RV. Right anterior oblique projection on coronary angiography showing evidence of coronary-cavitary fistula (arrow) (C). RV: right ventricle; LV: left ventricle; ADA: anterior descending artery.

Endomyocardial biopsy (EBM) is the gold standard method for the diagnosis of rejection after cardiac transplantation. Complications associated with the procedure are rare but can occur in about 8% of the cases. We describe a case of an uncommon complication probably associated with multiple EBMs in a transplanted patient. A 54-year-old male patient underwent orthotopic cardiac transplantation due to idiopathic dilated cardiomyopathy. Over the next ten months, the patient was submitted to eight EMBs and required pulse therapy on two occasions. In the routine outpatient evaluation, continuous systo diastolic murmur was observed in the left lower parasternal border and significant worsening of renal function. A transthoracic echocardiogram was performed, showing high velocity, right-sided apical flow on the color flow mapping and a significant dilation of the anterior descending coronary artery (ADA). The ADA presented significant dilatation with signs of communication with the right ventricle in the apical region, suggesting the diagnosis of coronary fistula (Figure 1A and B). Coronary angiography revealed aneurysmal ADA and vascular remodeling due to hyperflow, confirming the diagnosis of coronary-cavitary fistula (Figure 1C). The patient was submitted to percutaneous closure of the coronary fistula with Coil Vortex-18 device and presented good clinical evolution and improvement of renal function. It is important to consider the risks associated with EBM. Echocardiography is a valuable method for the evaluation of patients with suspected complications after the procedure. This case describes a rare complication after EBM and the diagnosis of which was possible by echocardiographic evaluation.

